# Supplementary material for: A Comparison of Evans Blue and 4-(p-Iodophenyl)butyryl Albumin Binding Moieties on an Integrin αvβ6 Binding Peptide
Source: Pharmaceutics. 2022 Mar 30;14(4):745. doi: 10.3390/pharmaceutics14040745 (PMC9025560; doi:10.3390/pharmaceutics14040745)
Supplement: Supplementary file 1 [file pharmaceutics-14-00745-s001.zip › pharmaceutics-1641549-supplementary.pdf]

pharmaceutics

Supplementary Materials: A Comparison of Evans Blue and 4-(*p*-Iodophenyl)butyryl Albumin Binding Moieties on an Integrin $\alpha_v\beta_6$ Binding Peptide

Ryan A. Davis, Sven H. Hausner, Rebecca Harris, and Julie L. Sutcliffe

Table S1. Table of Contents.

Title	Page#
RP-HPLC solvent gradient methods A & B	S3
Schematic for the solid phase reaction of the EB-ABM **8** to peptidyl resin of DOTA-K(NH_2)-$\alpha_v\beta_6$-BP to produce DOTA-EB-$\alpha_v\beta_6$-BP **1** after cleavage	S4
Pictorial of the reaction of **8** with peptidyl resin of DOTA-K(NH_2)-$\alpha_v\beta_6$-BP	S4
RP-HPLC of DOTA-EB-$\alpha_v\beta_6$-BP **1**	S5
MALDI-TOF of DOTA-EB-$\alpha_v\beta_6$-BP **1**	S5
Radio-RP-HPLC of [^{64}Cu]Cu DOTA-EB-$\alpha_v\beta_6$-BP, [^{64}Cu]**1**	S6
Co-injection radio-RP-HPLC of [^{64}Cu]Cu/[^{Nat}Cu]Cu DOTA-EB-$\alpha_v\beta_6$-BP, [^{Nat}Cu]**1** & [^{64}Cu]**1**	S6
MALDI-TOF of [^{Nat}Cu]Cu DOTA-EB-$\alpha_v\beta_6$-BP, [^{Nat}Cu]**1**	S7
RP-HPLC of DOTA-IP-$\alpha_v\beta_6$-BP **2**	S8
MALDI-TOF of DOTA-IP-$\alpha_v\beta_6$-BP **2**	S8
Radio-RP-HPLC of [^{64}Cu]Cu DOTA-IP-$\alpha_v\beta_6$-BP, [^{64}Cu]**2**	S9
Co-injection radio-RP-HPLC of [^{64}Cu]Cu/[^{Nat}Cu]Cu DOTA-IP-$\alpha_v\beta_6$-BP, [^{Nat}Cu]**2** & [^{64}Cu]**2**	S9
MALDI-TOF of [^{Nat}Cu]Cu DOTA-IP-$\alpha_v\beta_6$-BP, [^{Nat}Cu]**2**	S10
RP-HPLC of DOTA-EB-ABM **3**	S11

Table S2. Table of Contents.

Title	Page#
MALDI-TOF of DOTA-EB-ABM **3**	S11
Radio-RP-HPLC of [^{64}Cu]Cu DOTA-EB-ABM, [^{64}Cu]**3**	S12
Co-injection radio-RP-HPLC of [^{64}Cu]Cu/[^{Nat}Cu]Cu DOTA-EB-ABM, [^{Nat}Cu]**3** & [^{64}Cu]**3**	S12
MALDI-TOF of [^{Nat}Cu]Cu DOTA-EB-ABM, [^{Nat}Cu]**3**	S13
RP-HPLC of DOTA-IP-ABM **4**	S14
MALDI-TOF of DOTA-IP-ABM **4**	S14
Radio-RP-HPLC of [^{64}Cu]Cu DOTA-IP-ABM, [^{64}Cu]**4**	S15
Co-injection radio-RP-HPLC of [^{64}Cu]Cu/[^{Nat}Cu]Cu DOTA-IP-ABM, [^{Nat}Cu]**4** & [^{64}Cu]**4**	S15
MALDI-TOF of [^{Nat}Cu]Cu DOTA-IP-ABM, [^{Nat}Cu]**4**	S16
RP-HPLC of compound **6**	S17
ESI-FTMS of compound **6**	S17
RP-HPLC of EB-ABM **8**	S18
ESI-FTMS of EB-ABM **8**	S18
^{1}H NMR of EB-ABM **8**	S19
COSY NMR of EB-ABM **8**	S19
Biodistribution of compound [^{64}Cu]**1**	S20
Biodistribution of compound [^{64}Cu]**2**	S21
Tumor-to-Organ ratios from 4 h to 72 h p.i. of [^{64}Cu]**1** & [^{64}Cu]**2**	S22
Blocking biodistribution of compounds [^{64}Cu]**1** & [^{64}Cu]**2**	S23
Cell binding assay of compounds [^{64}Cu]**3** & [^{64}Cu]**4**	S24

Biodistribution of compounds [^{64}Cu]**3** & [^{64}Cu]**4** S25

Summary of Tumor-to-Organ ratios at 4 h for [^{64}Cu]**1-4** S26

Graph of Summarized Tumor-to-Organ ratios at 4 h for [^{64}Cu]**1-4** S26

Table S3. RP-HPLC methods.

Semi-preparative HPLC Gradient (3 mL/min)		
Time (minutes)	%Acetonitrile	% Water with 0.05% TFA
0	9%	91%
2	9%	91%
5	20%	80%
7	20%	80%
25	91%	9%
30	9%	91%

RP-HPLC solvent gradient method A. Semi-preparative purification conditions. Flow rate 3 mL/min. Column: Phenomenex Jupiter (250 x 10 mm, 10 µm)

Analytical HPLC Solvent Gradient (1 mL/min)		
Time (minutes)	%Acetonitrile	% Water with 0.05% TFA
0	9%	91%
2	9%	91%
32	81%	19%

RP-HPLC solvent gradient method B. Analytical quality control method for all compounds. Flow rate 1.5 mL/min. Column: Phenomenex Jupiter Proteo (250 mm x 4.6 mm , 4 µm)

Schematic for the solid phase reaction of the EB-ABM **8** to peptidyl resin of DOTA-K(NH$_2$)-$_v$ $_6$-BP to produce DOTA-EB-$\alpha_v\beta_6$-BP **1** after cleavage.

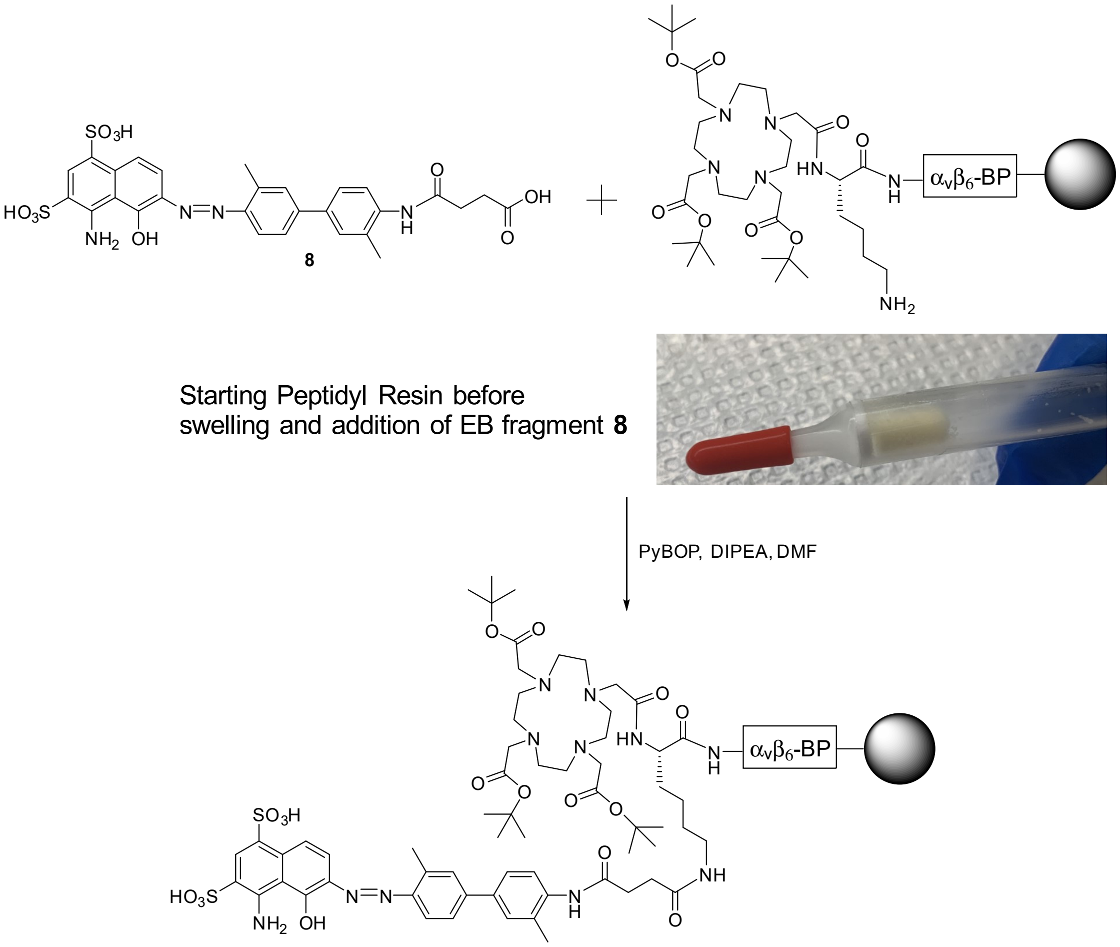

Pictorial of the reaction of **8** with peptidyl resin of DOTA-K(NH$_2$)-$\alpha_v\beta_6$-BP. **A**. Reaction of **8** and peptidyl resin. **B**. Rinsing of peptidyl resin after coupling of **8**. **C**. Peptidyl resin of DOTA-EB-$_v$ $_6$-BP **1**.

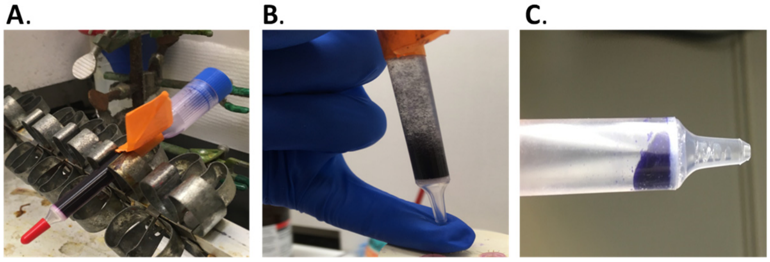

Figure S4. Schematic for solid phase reaction of EB-ABM **8** to peptidyl resin of DOTA-K(NH$_2$-$\alpha_v\beta_6$-BP to produce DOTA-EB-$\alpha_v\beta_6$-BP **1** after cleavage and pictorial of the reaction of **8** with peptidyl resin of DOTA-K(NH$_2$)-$\alpha_v\beta_6$-BP.

RP-HPLC of DOTA-EB-$\alpha_v\beta_6$-BP **1**
Retention time 17.22 min, UV-vis (220 nm)

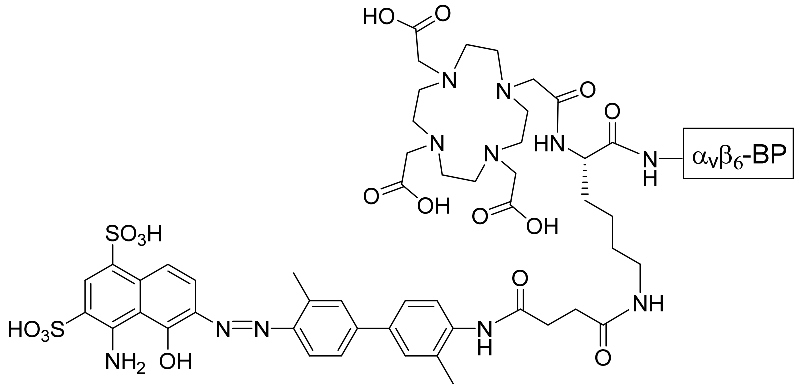

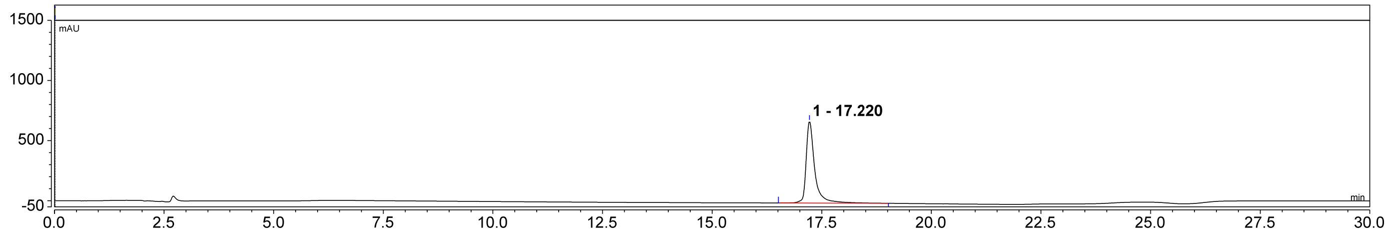

MALDI-TOF of DOTA-EB-$\alpha_v\beta_6$-BP **1**
m/z: $[M+Na]^+$ calc'd for $C_{261}H_{460}N_{46}NaO_{102}S_2$ 5958.1556; found 5958.1756.

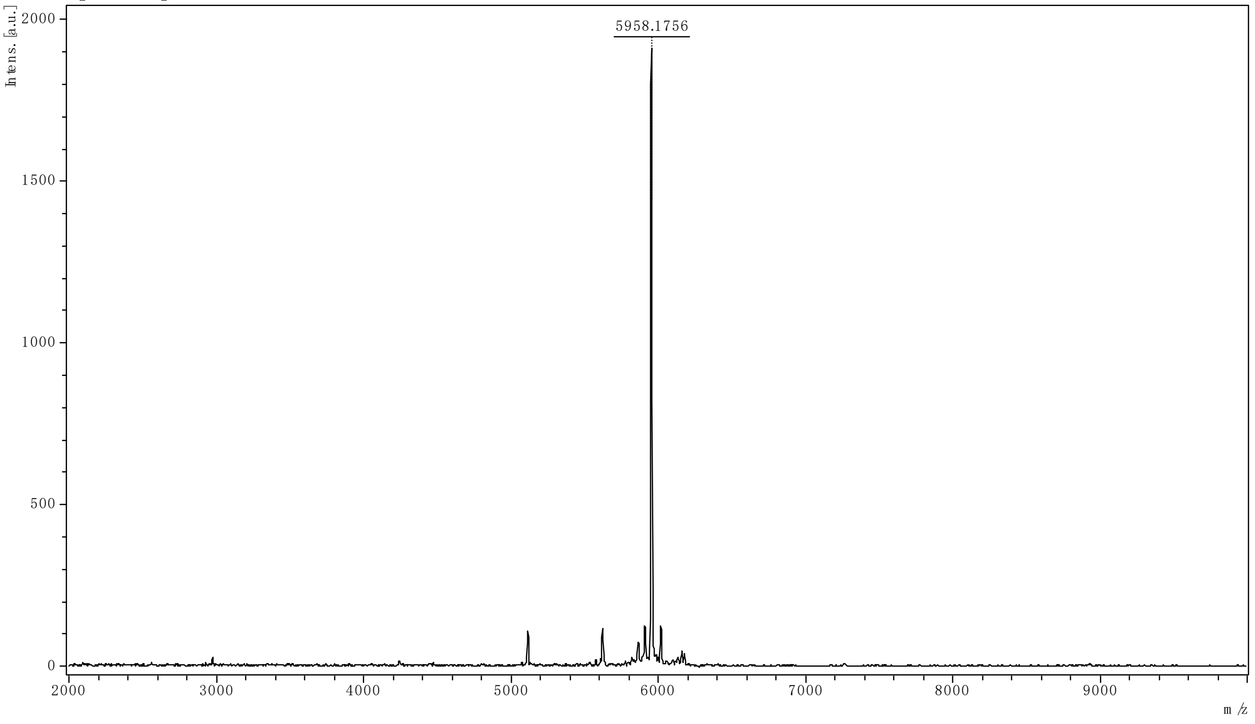

Figure S5. RP-HPLC and MALDI-TOF of DOTA-EB-$\alpha_v\beta_6$-BP **1.**

Radio-RP-HPLC of [^{64}Cu]Cu DOTA-EB-$\alpha_v\beta_6$-BP, [^{64}Cu]**1**
Retention time 19.05 min, Gamma-detector

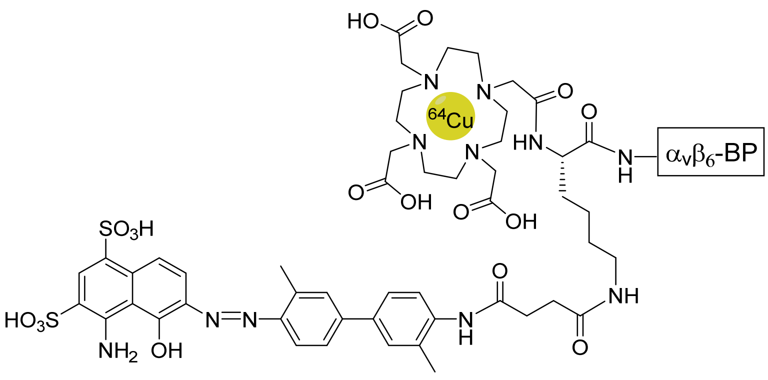

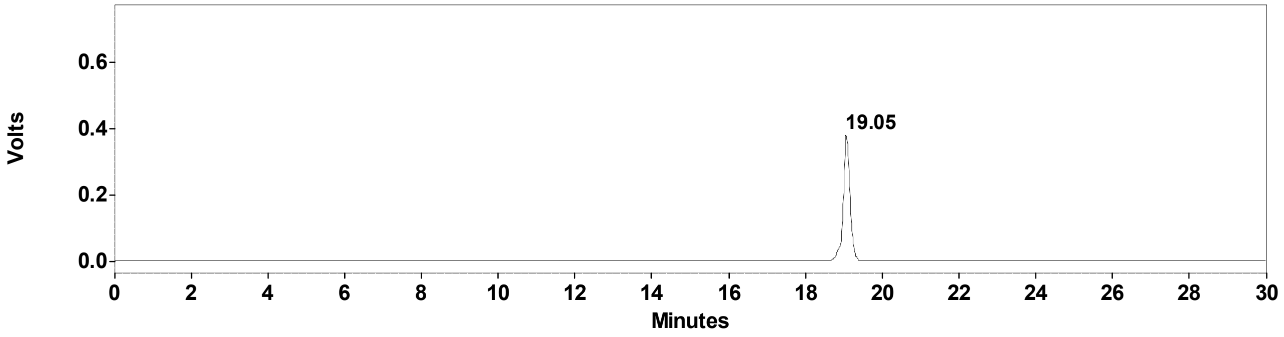

Co-injection radio-RP-HPLC of [^{64}Cu]Cu/[^{Nat}Cu]Cu DOTA-EB-$\alpha_v\beta_6$-BP, [^{Nat}Cu]**1** & [^{64}Cu]**1**
Black: UV-vis (220 nm), Red: Gamma-detector

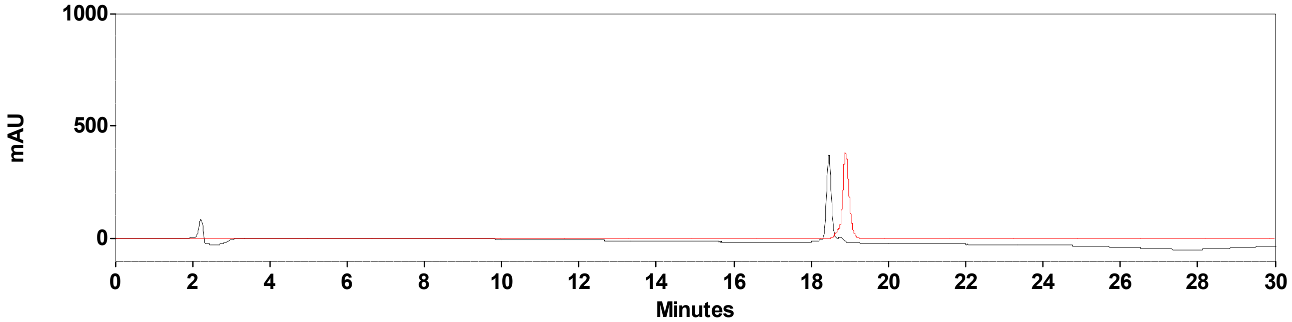

Figure S6. Radio-RP-HPLC of [^{64}Cu]**1** and co-injection radio-RP-HPLC of [^{Nat}Cu]**1** and [^{64}Cu]**1.**

MALDI-TOF of [NatCu]Cu DOTA-EB-$\alpha_v\beta_6$-BP, [NatCu]**1**

m/z: [M+Cu]$^+$ calc'd for $C_{261}H_{459}CuN_{46}NaO_{102}S_2$ 6020.0773; found 6020.0761.

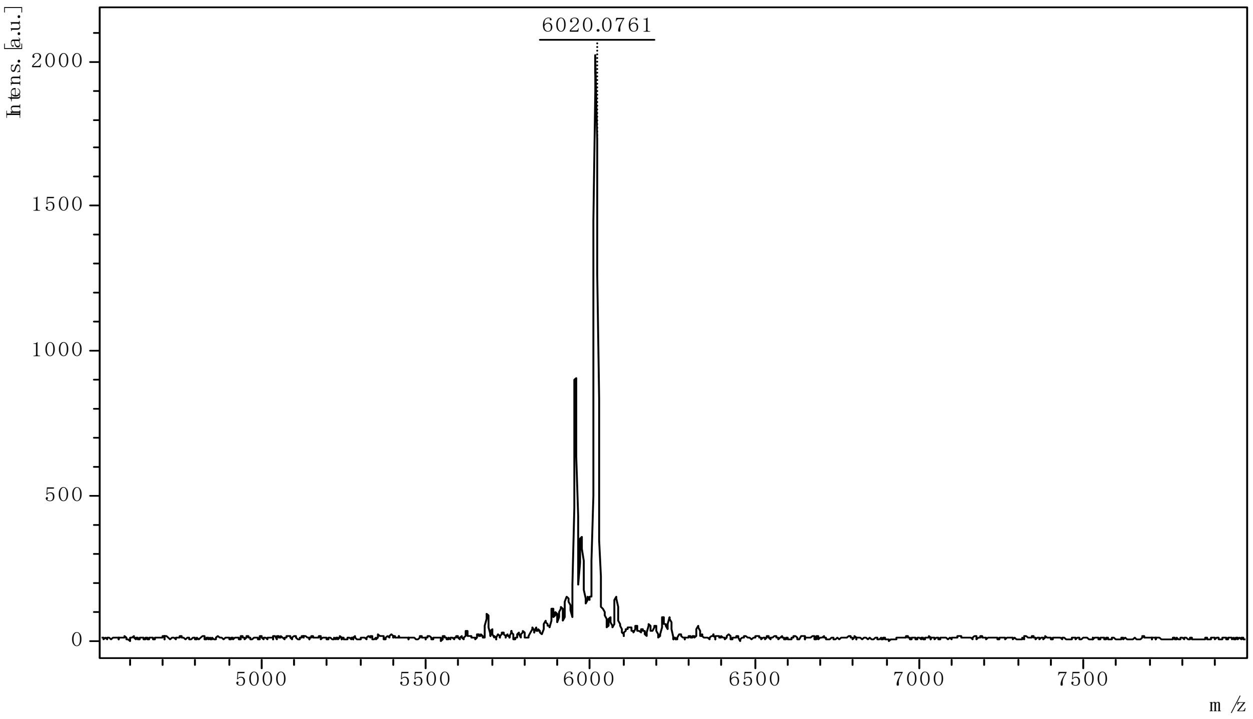

Figure S7. MALDI-TOF of [NatCu]**1.**

RP-HPLC of DOTA-IP-$\alpha_v\beta_6$-BP **2**
Retention time 17.07 min, UV-vis (220 nm)

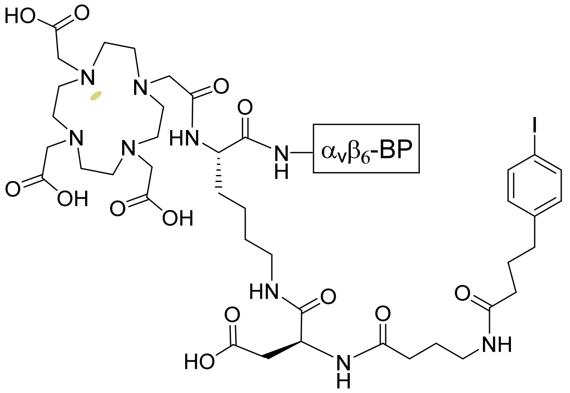

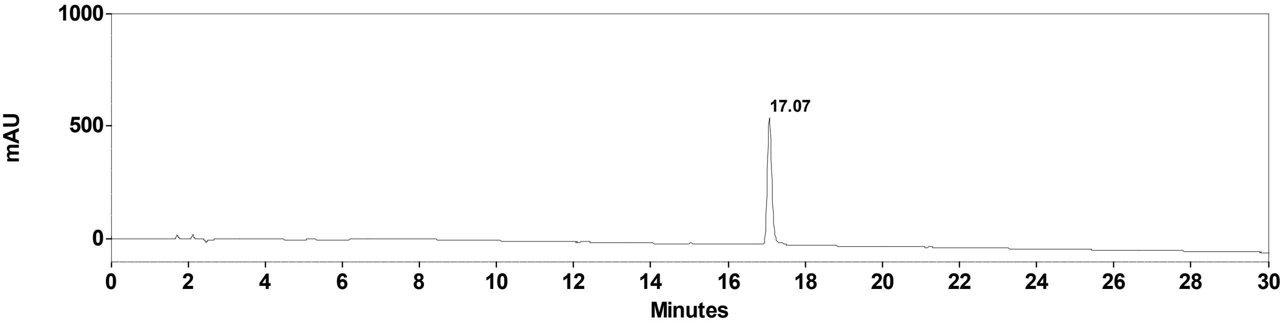

MALDI-TOF of DOTA-IP-$\alpha_v\beta_6$-BP **2**
m/z: $[M+H]^+$ calc'd for $C_{251}H_{458}IN_{44}O_{98}$ 5786.1314; found 5786.1209.

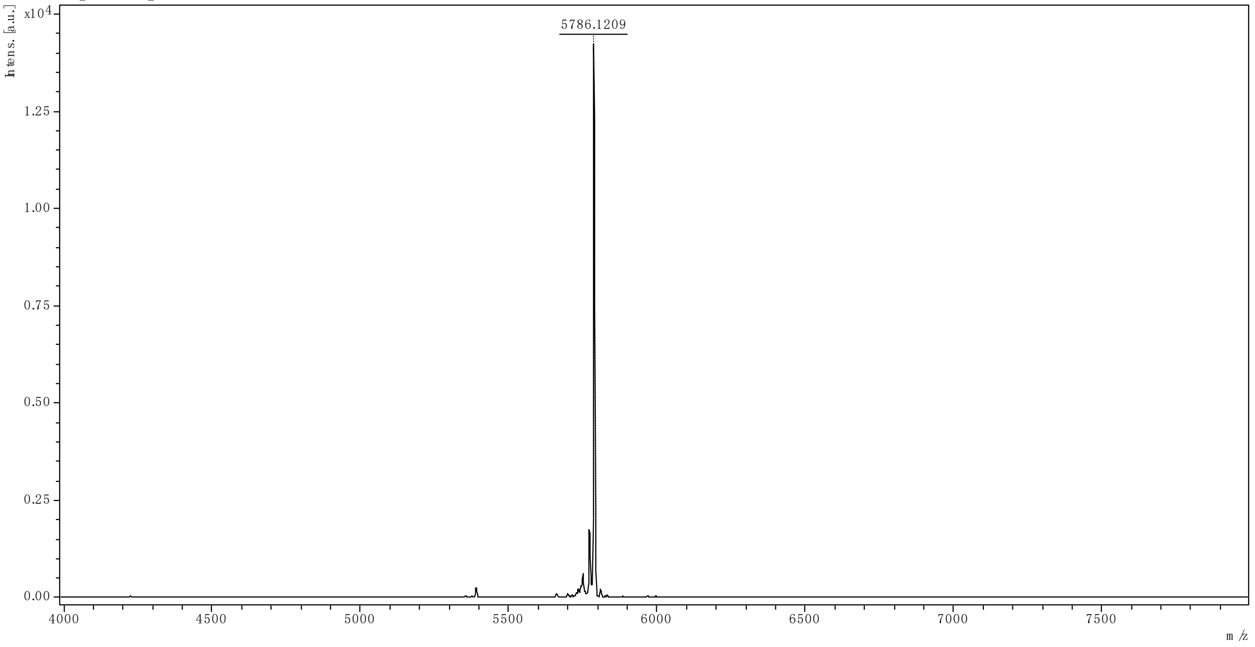

Figure S8. RP-HPLC and MALDI-TOF of DOTA-IP-$\alpha_v\beta_6$-BP **2.**

Radio-RP-HPLC of [^{64}Cu]Cu DOTA-IP- $_{v}$ $_{6}$-BP, [^{64}Cu]**2**
Retention time 18.68 min, Gamma-detector

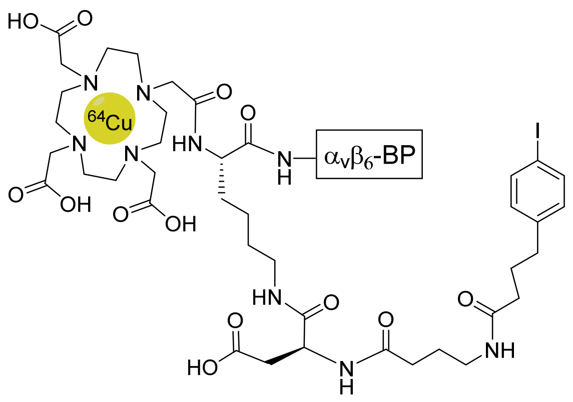

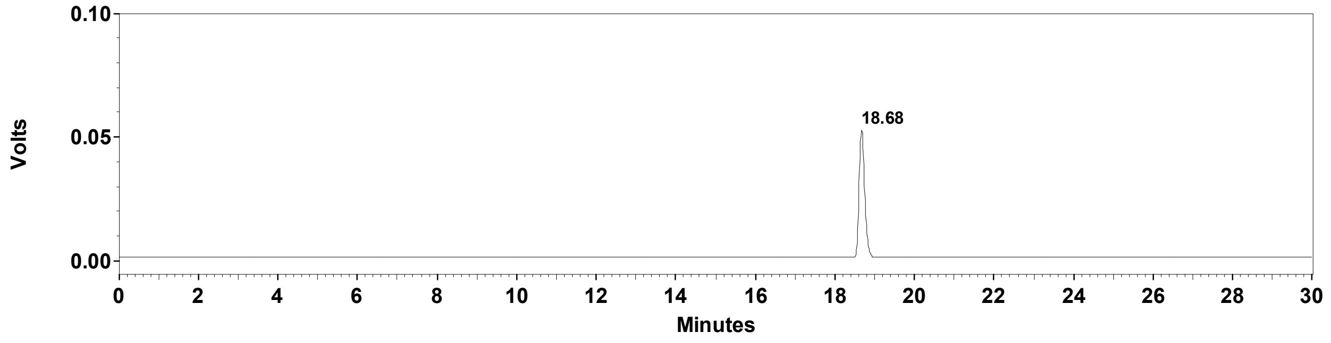

Co-injection radio-RP-HPLC of [^{64}Cu]Cu/[^{Nat}Cu]Cu DOTA-IP- $_{v}$ $_{6}$-BP, [^{Nat}Cu]**2** & [^{64}Cu]**2**
Black: UV-vis (220 nm), Red: Gamma-detector

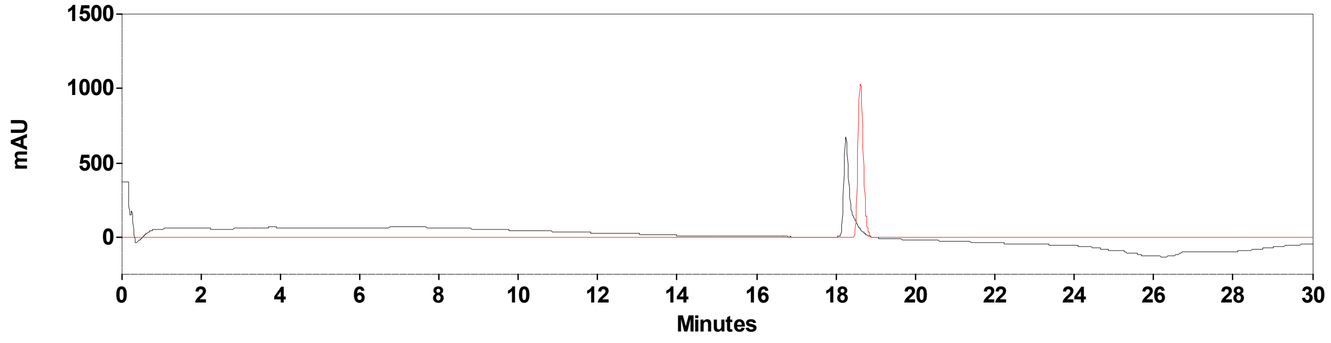

Figure S9. Radio-RP-HPLC of [^{64}Cu]**2** and co-injection radio-RP-HPLC of [^{Nat}Cu]**2** and [^{64}Cu]**2.**

MALDI-TOF of [NatCu]Cu DOTA-IP-$\alpha_v\beta_6$-BP, [NatCu]**2**
m/z: [M+Cu]$^+$ calc'd for $C_{251}H_{456}CuIN_{44}NaO_{98}$ 5868.0284; found 5868.0369.

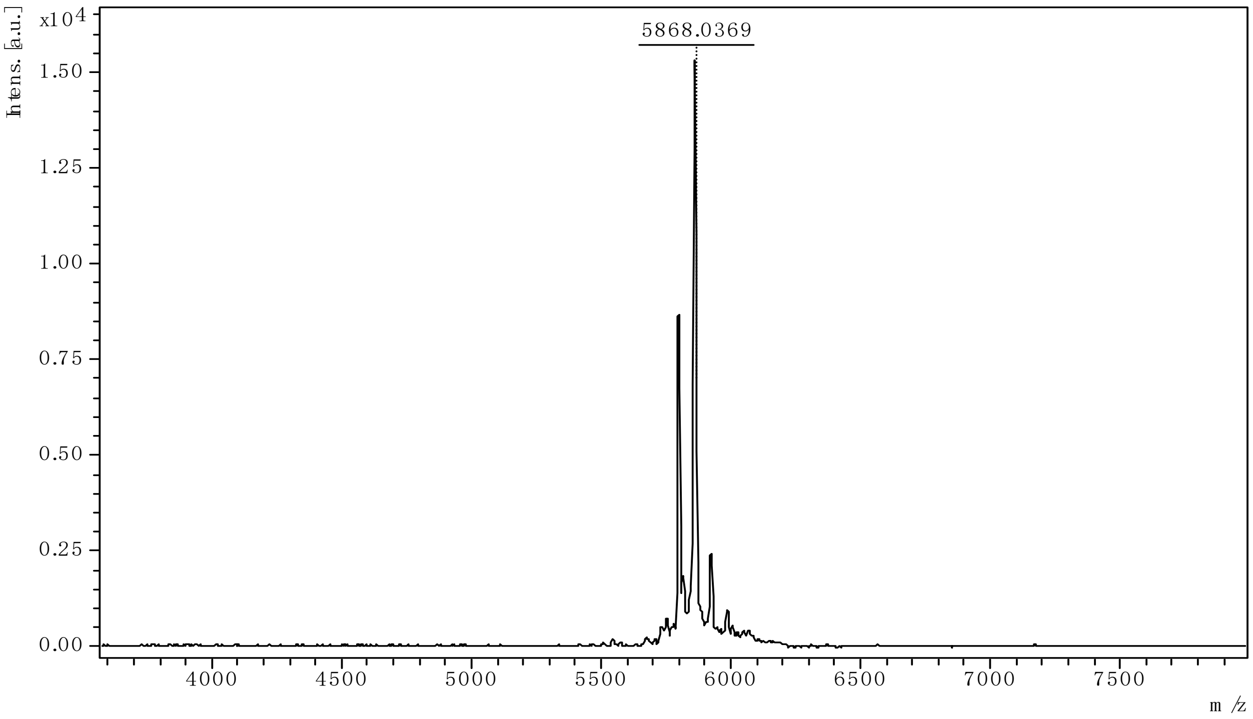

Figure S10. MALDI-TOF of [NatCu]**2.**

RP-HPLC of DOTA-EB-ABM **3**
Retention time 14.18 min, UV-vis (220 nm)

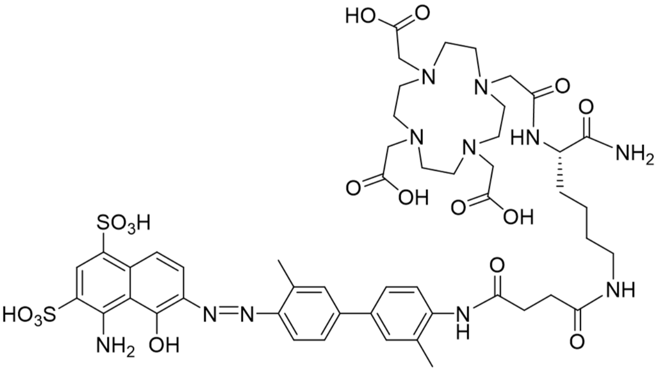

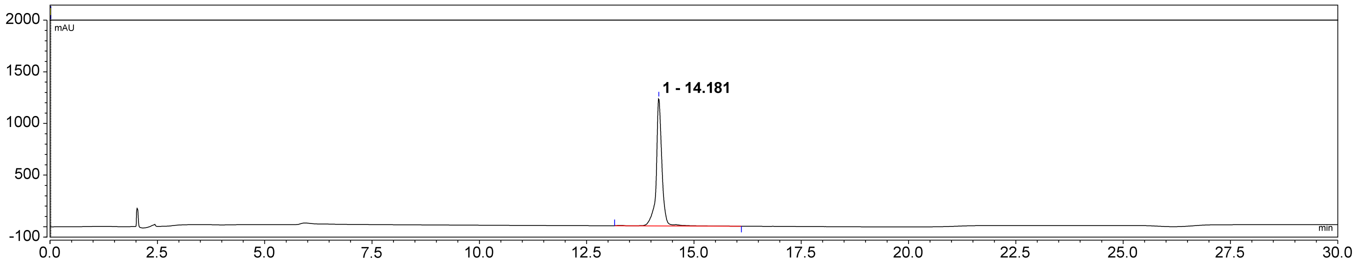

MALDI-TOF of DOTA-EB-ABM **3**
m/z: $[M+H]^+$ calc'd for $C_{50}H_{66}N_{11}O_{17}S_2$ 1156.4074; found 1156.4079.

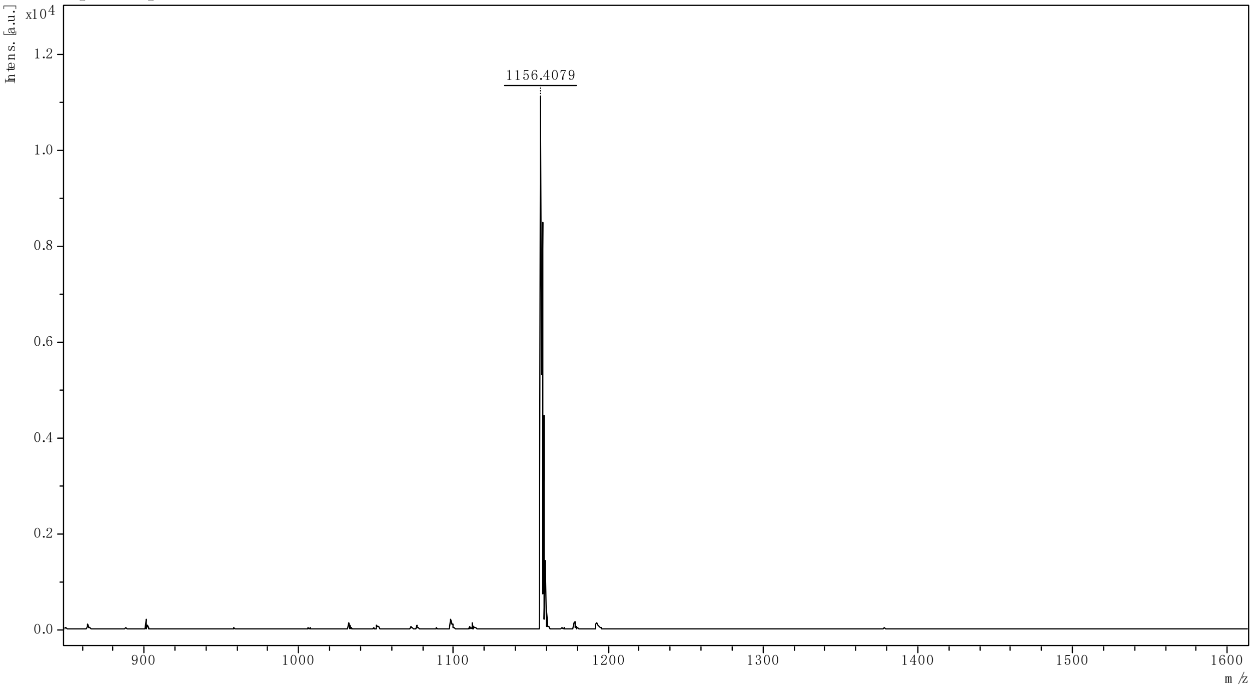

Figure S11. RP-HPLC and MALDI-TOF of DOTA-EB-ABM **3.**

Radio-RP-HPLC of [^{64}Cu]Cu DOTA-EB-ABM, [^{64}Cu]**3**
Retention time 17.01 min, Gamma-detector

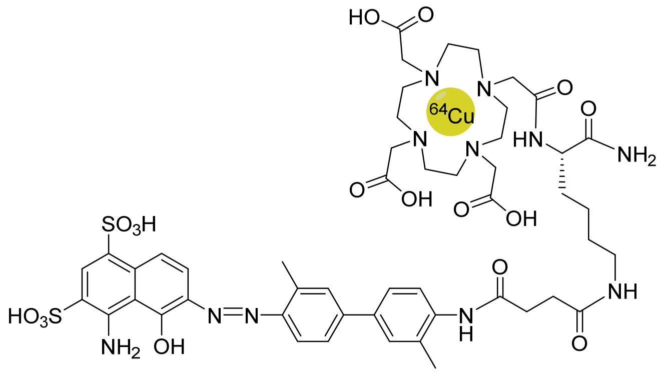

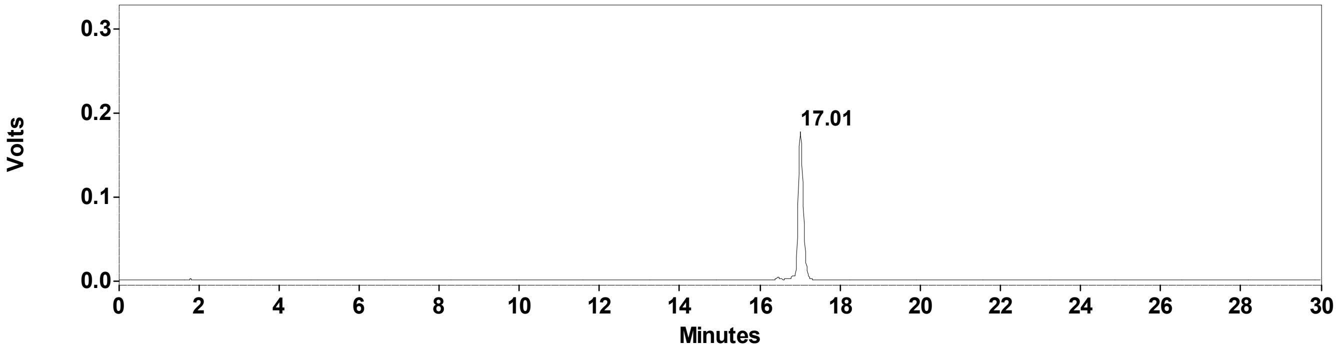

Co-injection radio-RP-HPLC of [^{64}Cu]Cu/[^{Nat}Cu]Cu DOTA-EB-ABM, [^{Nat}Cu]**3** & [^{64}Cu]**3**
Black: UV-vis (220 nm), Red: Gamma-detector

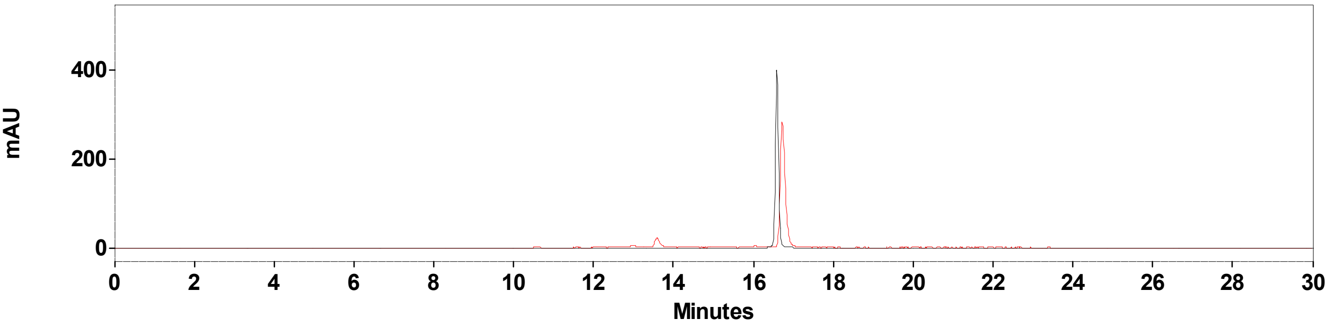

Figure S12. Radio-RP-HPLC of [^{64}Cu]**3** and co-injection radio-RP-HPLC of [^{Nat}Cu]**3** and [^{64}Cu]**3.**

MALDI-TOF of [NatCu]Cu DOTA-EB-ABM, [NatCu]**3**

m/z: [M+Cu]+ calc'd for $C_{50}H_{65}CuN_{11}O_{17}S_2$ 1218.3292; found 1218.3829.

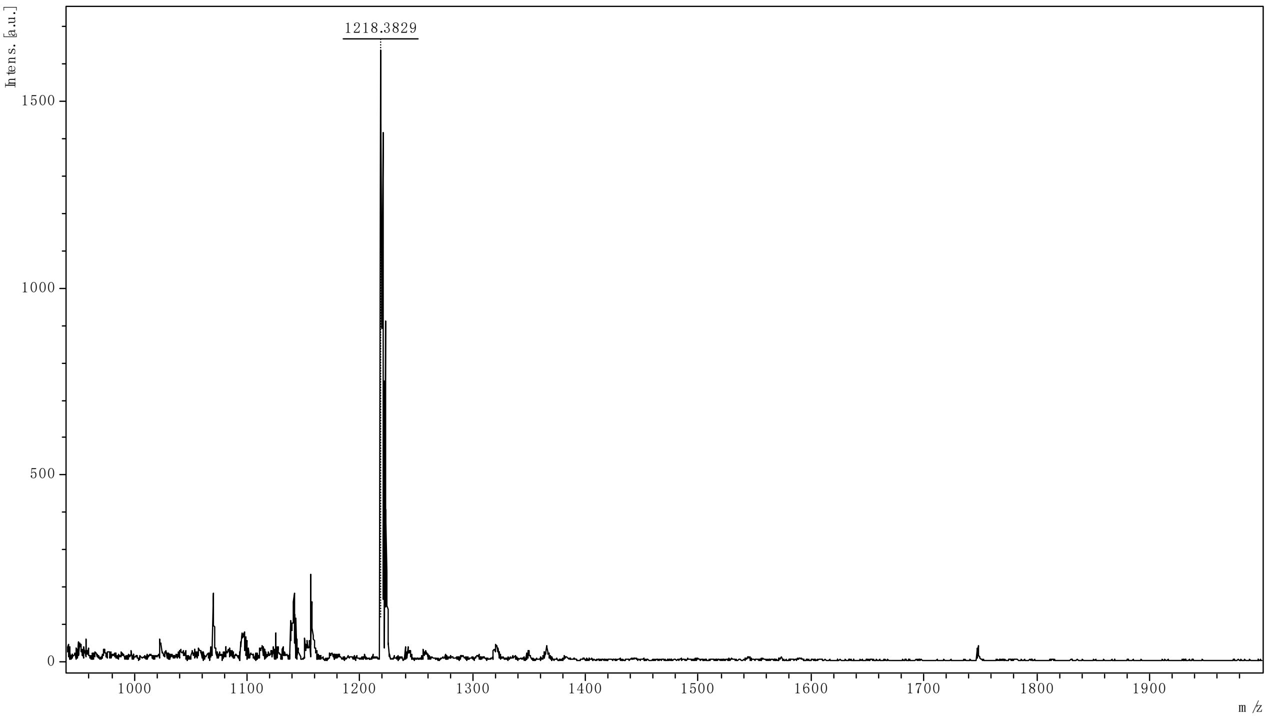

Figure S13. MALDI-TOF of [NatCu]**3.**

RP-HPLC of DOTA-IP-ABM (**4**)
Retention time 14.46 min, UV-vis (220 nm)

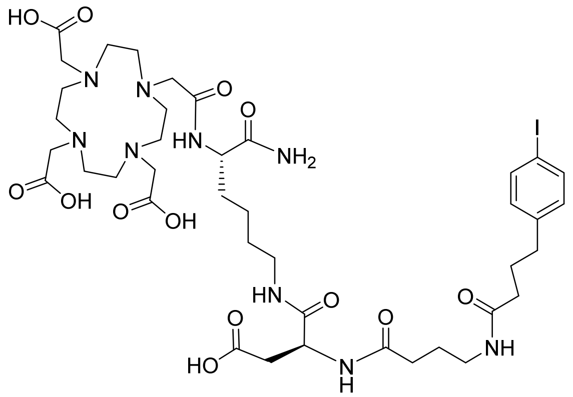

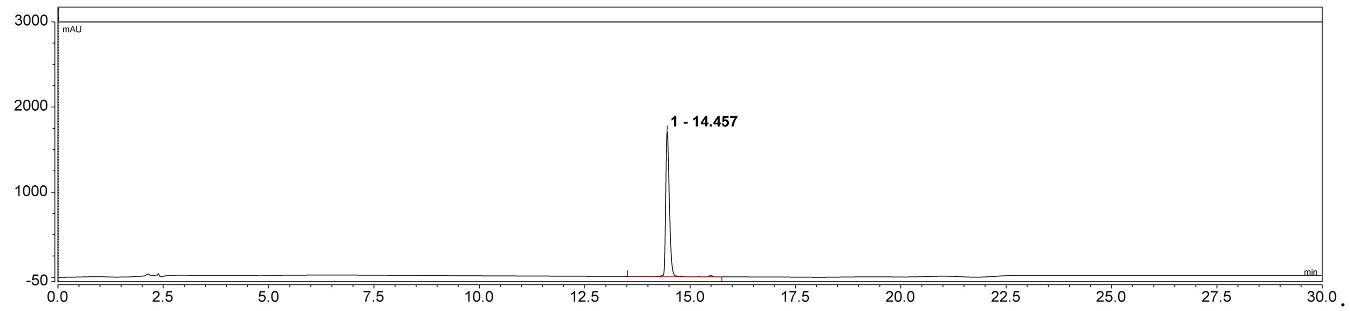

MALDI-TOF of DOTA-IP-ABM (**4**)
m/z: [M+H]+ calc'd for $C_{40}H_{63}IN_9O_{13}$ 1004.3585; found 1004.3590.

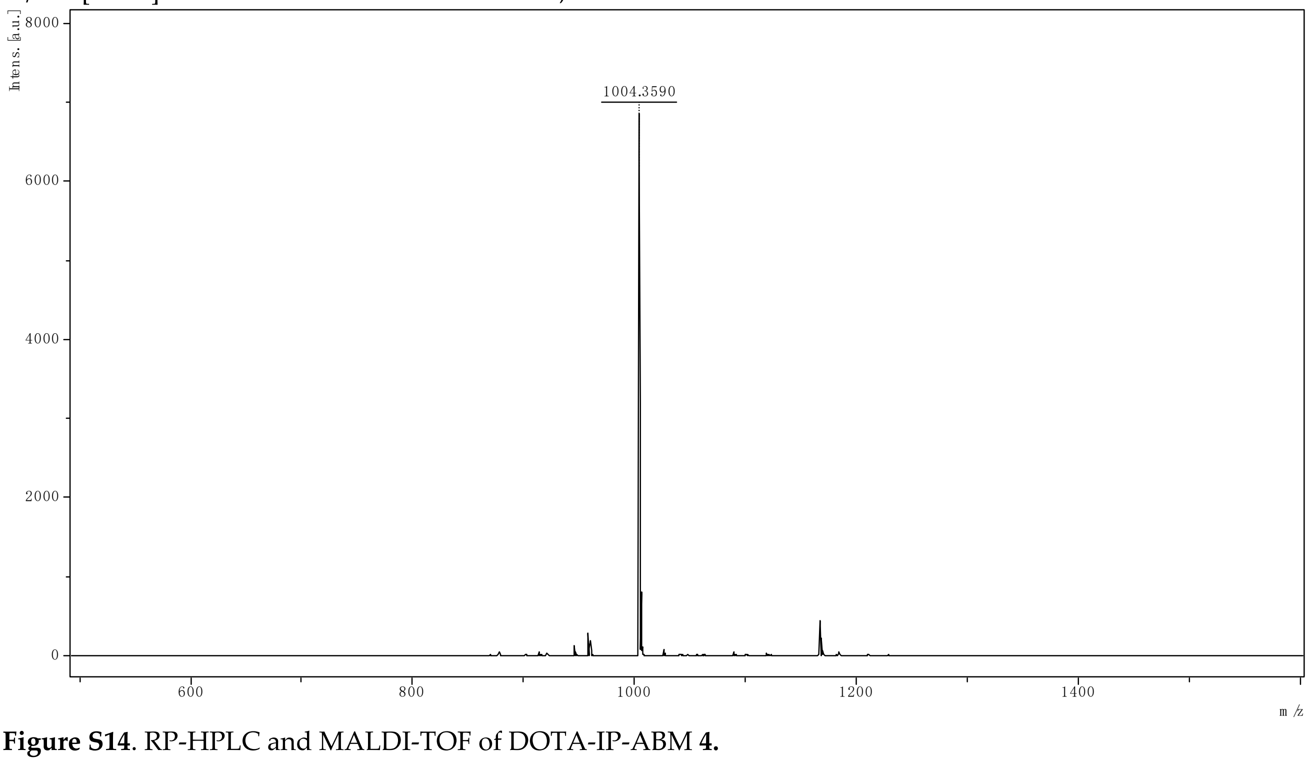

Figure S14. RP-HPLC and MALDI-TOF of DOTA-IP-ABM **4.**

Radio-RP-HPLC of [^{64}Cu]Cu DOTA-IP-ABM, [^{64}Cu]**4**
Retention time 16.73 min, Gamma-detector

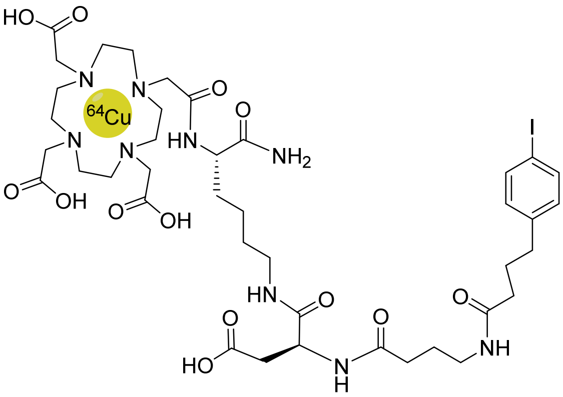

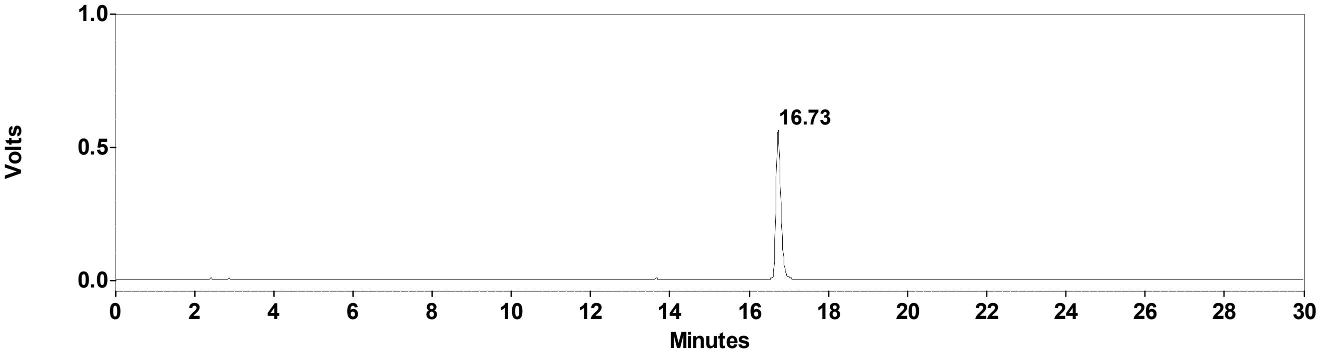

Co-injection radio-RP-HPLC of [^{64}Cu]Cu/[^{Nat}Cu]Cu DOTA-IP-ABM, [^{Nat}Cu]**4** & [^{64}Cu]**4**
Black: UV-vis (220 nm), Red: Gamma-detector

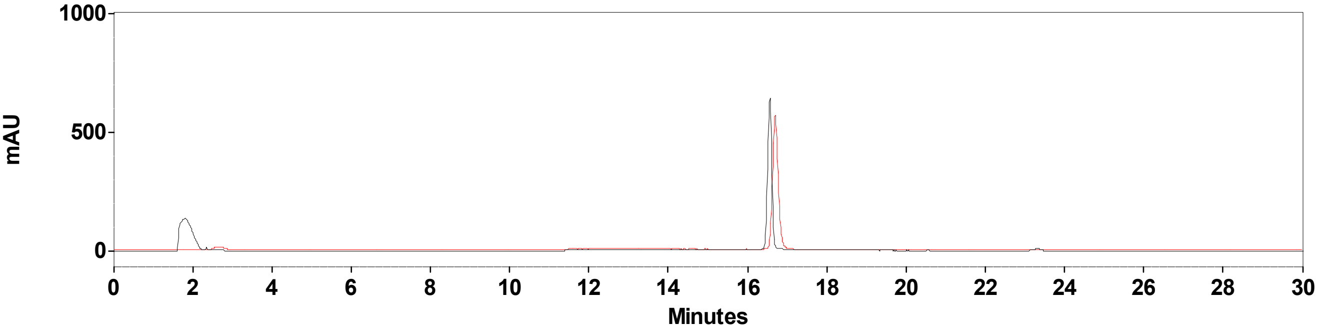

Figure S15. Radio-RP-HPLC of [^{64}Cu]**4** and co-injection radio-RP-HPLC of [^{Nat}Cu]**4** and [^{64}Cu]**4.**

MALDI-TOF of [^{Nat}Cu]Cu DOTA-IP-ABM, [^{Nat}Cu]**4**

m/z: [M+Cu]$^+$ calc'd for $C_{40}H_{62}CuIN_9O_{13}$ 1066.2802; found 1066.2690.

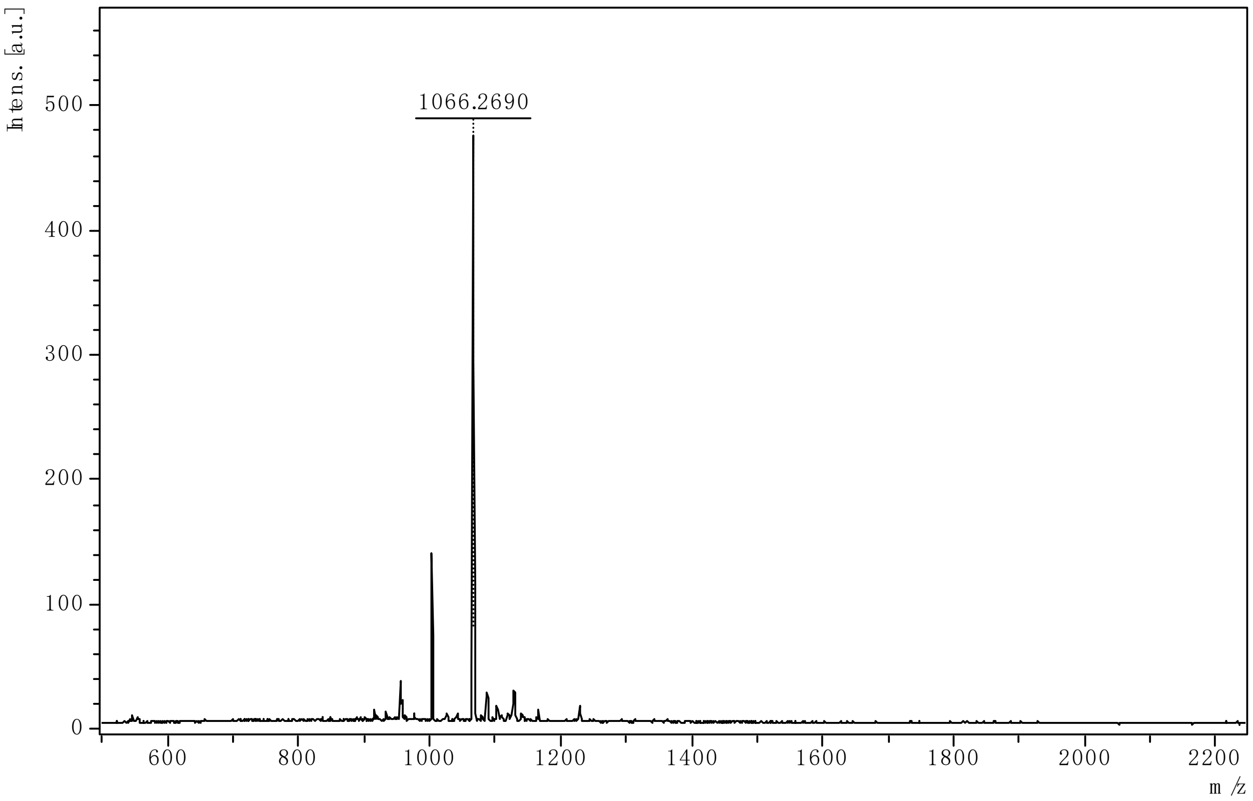

Figure S16. MALDI-TOF of [^{Nat}Cu]**4.**

RP-HPLC of compound **6**
Retention time 10.07 min, UV-vis (220 nm)

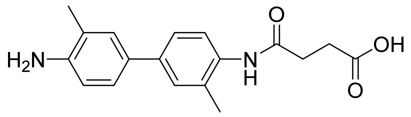

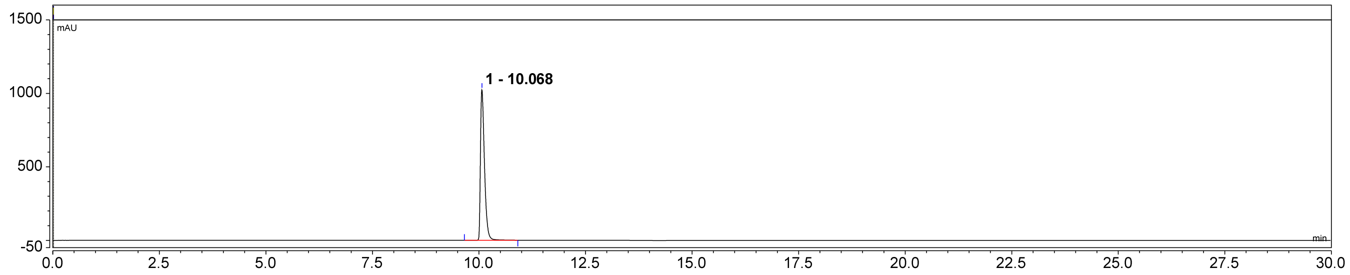

ESI-FTMS of compound **6**
m/z: $[M+H]^+$ calc'd for $C_{18}H_{21}N_2O_3$ 313.1547; found 313.1545.

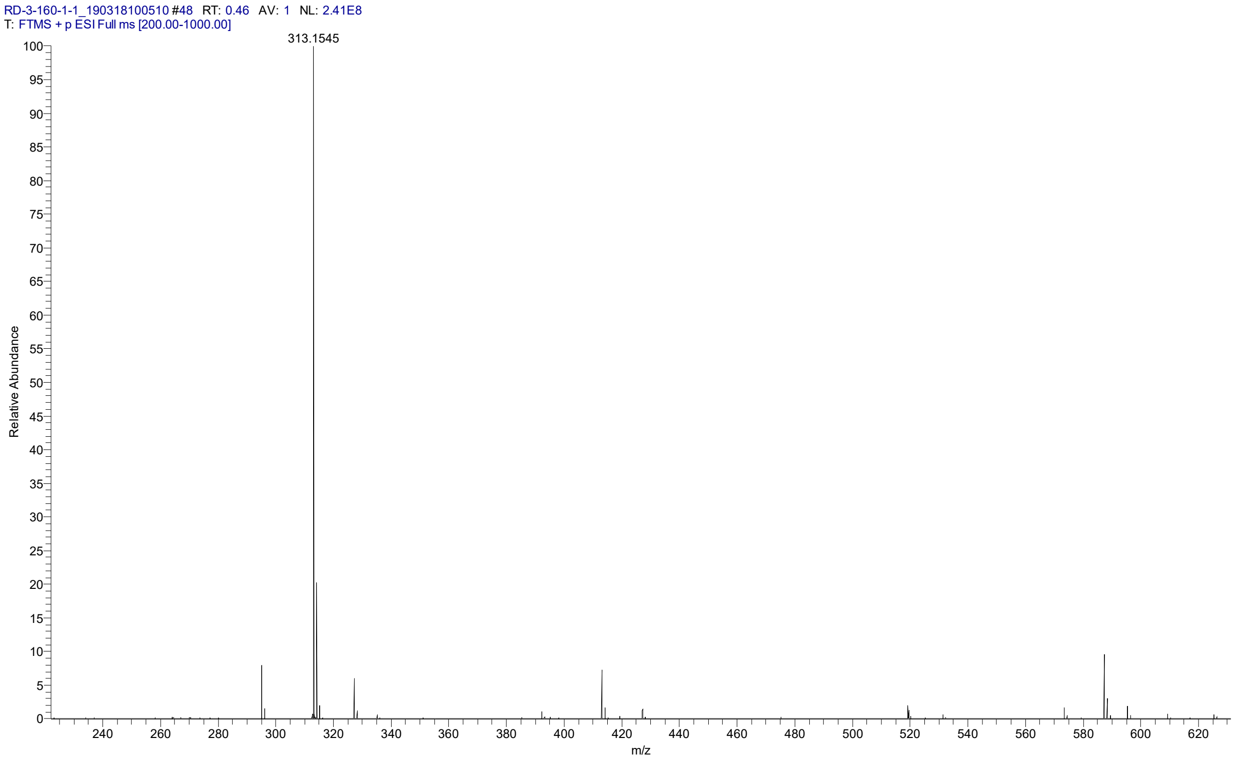

Figure S17. RP-HPLC and ESI-FTMS of compound **6.**

RP-HPLC of EB-ABM **8**
Retention time 17.72 min, UV-vis (220 nm)

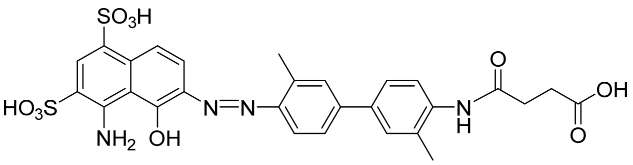

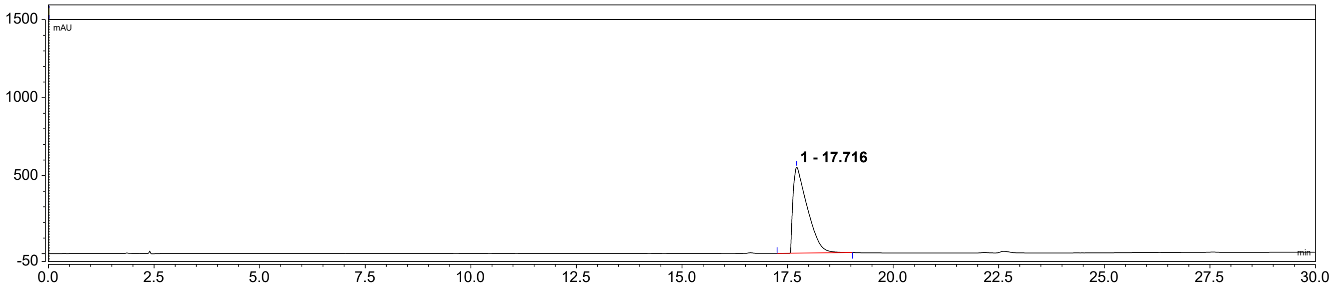

ESI-FTMS of EB-ABM **8**
m/z: [M+H]$^+$calc'd for $C_{28}H_{27}N_4O_{10}S_2$ 643.1163; found 643.1207.

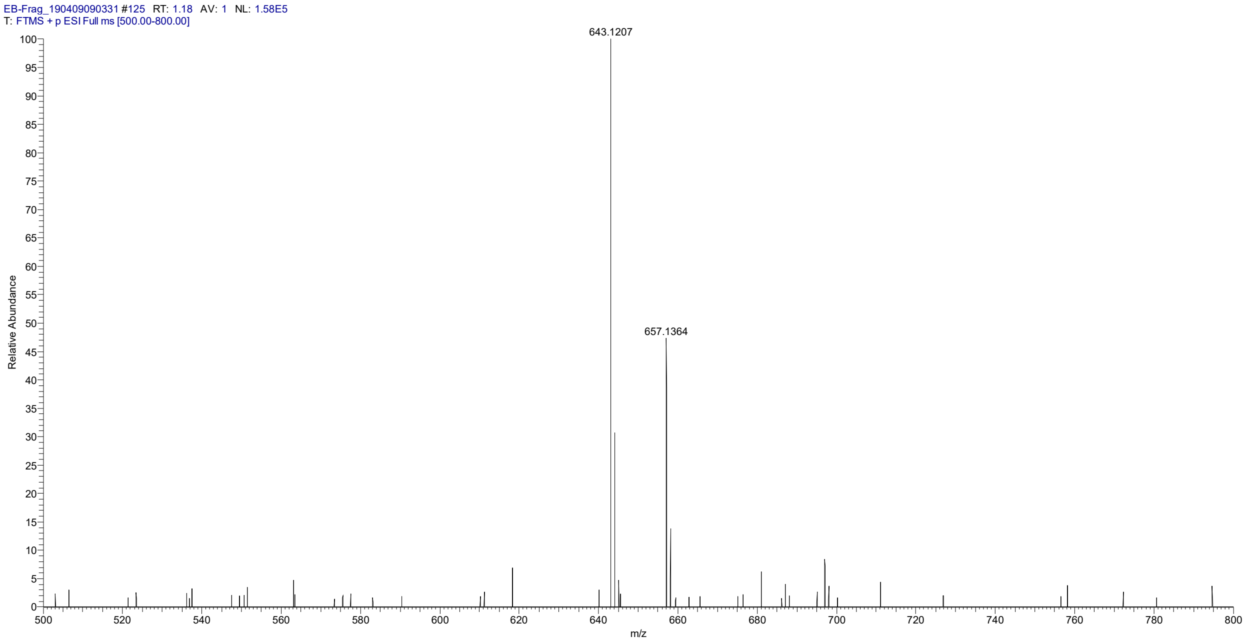

Figure S18. RP-HPLC and ESI-FTMS of EB-ABM **8.**

^1H NMR of EB-ABM **8**

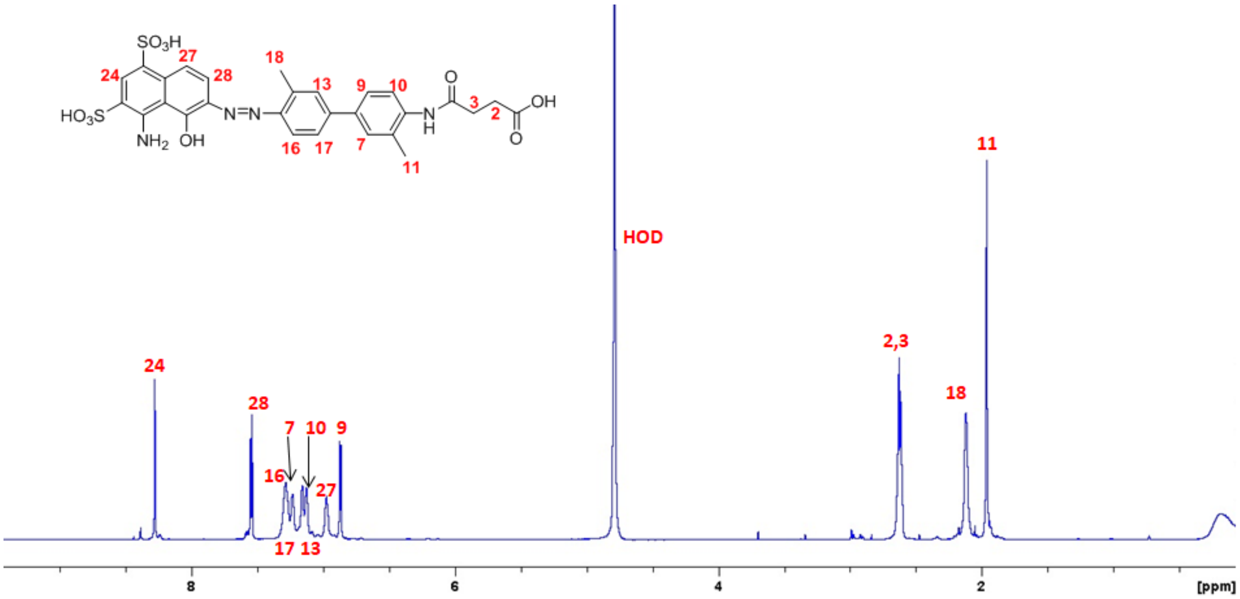

H′ NMR (800 MHz, D_2O) δ 8.28 (s, 1H), 7.55 (d, *J* = 9.4 Hz, 1H), 7.29–7.27 (m, 2H), 7.24-7.23 (m, 1H), 7.17-7.16 (m, 1H), 7.13-7.12 (m, 1H), 6.98-6.97 (m, 1H), 6.88 (d, 7.8 Hz, 1H), 2.64-2.61 (m, 4H), 2.12 (s, 3H), 1.96 (s, 3H).

COSY NMR of EB-ABM **8**

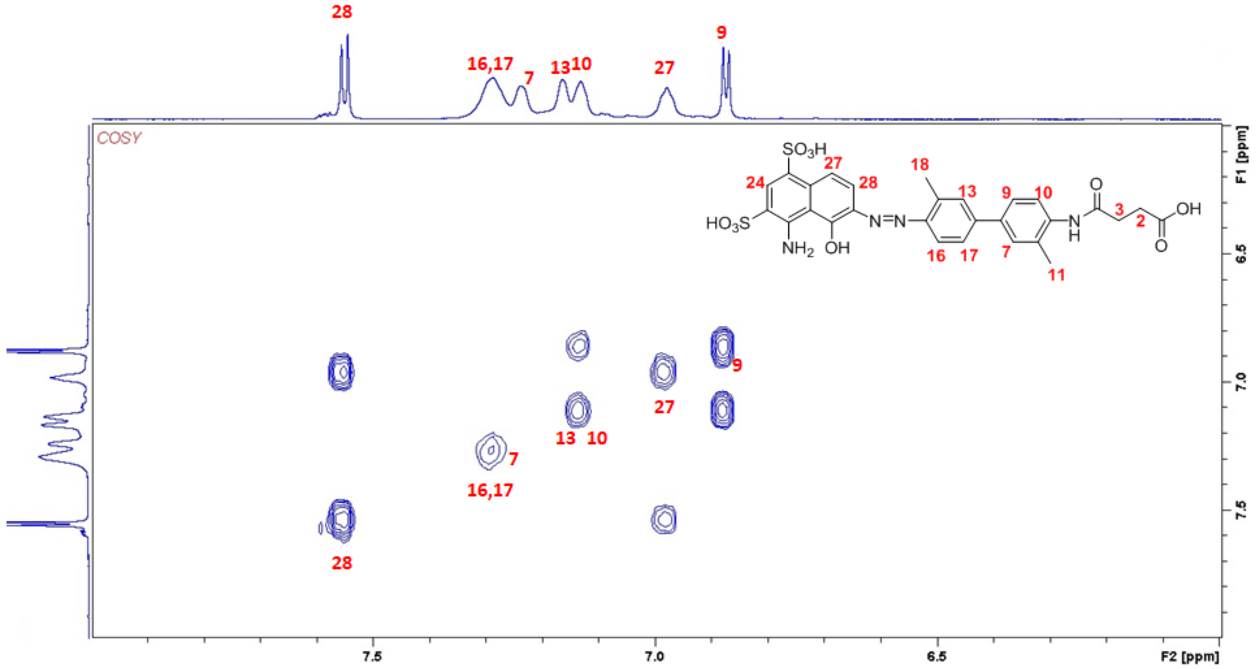

Figure S19. ^1H NMR and COSY of EB-ABM **8.**

Biodistribution of compound [^{64}Cu]**1**

Tissue	4 h	24 h	48 h	72 h
BxPC-3 Tumor	5.29 ± 0.59	5.03 ± 0.97*	4.00 ± 1.51	3.32 ± 0.46
Blood	1.30 ± 0.31	0.33 ± 0.01	0.33 ± 0.10	0.35 ± 0.08
Pancreas	0.46 ± 0.06	0.48 ± 0.03	0.41 ± 0.20	0.59 ± 0.12
Liver	1.64 ± 0.20	2.09 ± 0.10	2.59 ± 0.51	2.36 ± 0.51
Muscle	0.67 ± 0.10	0.50 ± 0.20	0.49 ± 0.07	0.44 ± 0.12
Kidneys	75.51 ± 7.26	40.50 ± 7.70	26.18 ± 5.89	19.97 ± 6.91
Gall Bladder	2.12 ± 0.51	1.85 ± 0.27	1.75 ± 0.61	1.75 ± 0.65
Heart	0.99 ± 0.08	0.92 ± 0.05	1.02 ± 0.17	1.15 ± 0.20
Lung	2.71 ± 0.57	2.79 ± 1.62	2.33 ± 0.32	1.88 ± 0.11
Spleen	0.32 ± 0.28	0.56 ± 0.16	0.50 ± 0.18	0.58 ± 0.16
Stomach	6.41 ± 0.64	4.18 ± 0.54	3.02 ± 0.52	2.02 ± 0.16
Sm. Intestines	4.72 ± 0.55	2.78 ± 0.31	1.85 ± 0.07	1.38 ± 0.18
Lg. Intestines	4.13 ± 0.10	3.46 ± 0.45	2.65 ± 0.05	1.95 ± 0.29
Skin	1.56 ± 0.34	1.08 ± 0.30	1.12 ± 0.18	0.88 ± 0.12
Bone	0.44 ± 0.04	0.34 ± 0.13	0.30 ± 0.08	0.25 ± 0.13
Brain	0.09 ± 0.02	0.09 ± 0.01	0.12 ± 0.02	0.16 ± 0.04
Urine	19.64 ± 10.53	5.01 ± 0.29*	3.92 ± 1.05	3.17 ± 1.58
Fecal Matter	3.03 ± 0.67	2.85 ± 0.55	2.34 ± 0.89	1.81 ± 0.74

Biodistribution data for compound [^{64}Cu]**1** in mice bearing BxPC-3 xenograft tumors. Organ uptake is expressed as percent of injected dose per gram of tissue (% ID/g, n = 3, animals/time point). (*-n = 2, one mouse had no tumor and provided no urine)

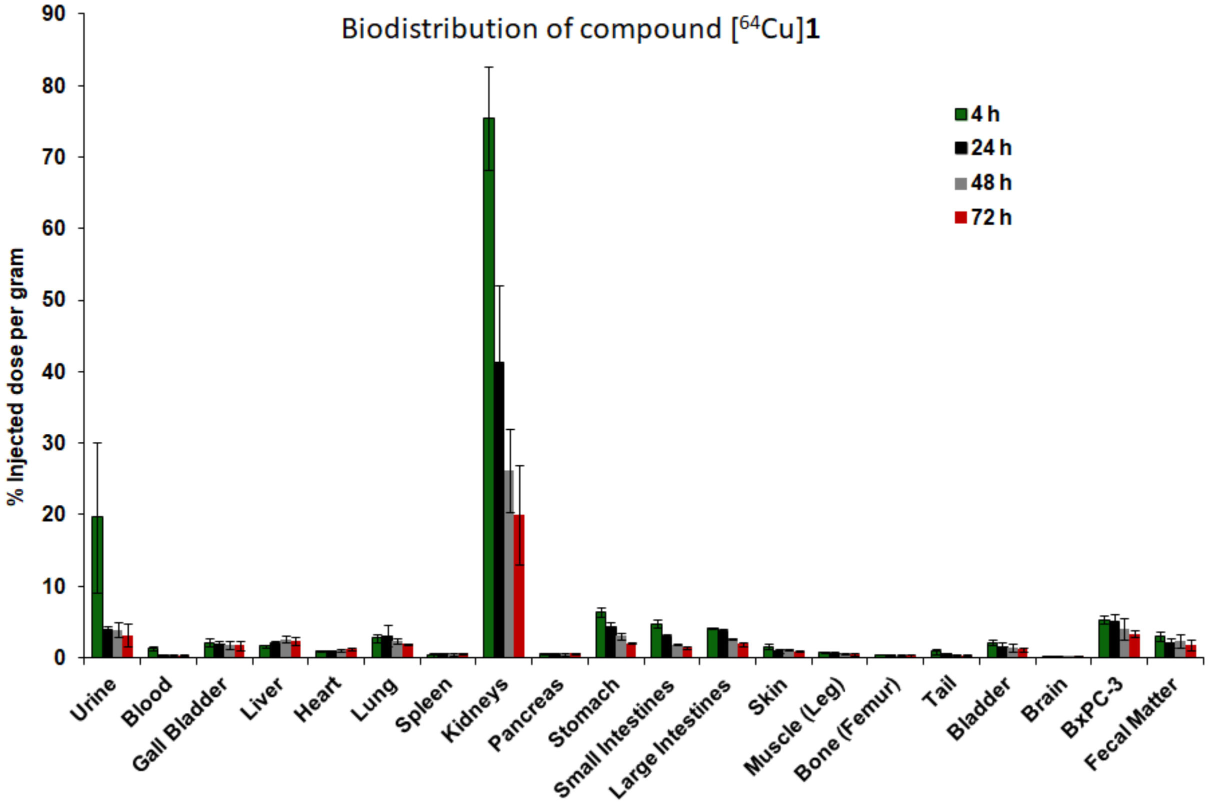

Figure S20. Biodistribution of [^{64}Cu]**1.**

Biodistribution of compound [^{64}Cu]**2**

Tissue	4 h	24 h	48 h	72 h
BxPC-3 Tumor	7.60 ± 0.43	4.41 ± 1.25	4.09 ± 1.28	4.91 ± 1.19
Blood	1.21 ± 0.16	0.20 ± 0.04	0.20 ± 0.04	0.17 ± 0.001
Pancreas	1.04 ± 0.09	0.78 ± 0.01	0.64 ± 0.08	0.57 ± 0.06
Liver	1.58 ± 0.26	1.62 ± 0.20	1.71 ± 0.21	1.30 ± 0.13
Muscle	0.99 ± 0.30	0.78 ± 0.22	0.63 ± 0.07	0.76 ± 0.24
Kidneys	33.56 ± 5.39	21.95 ± 1.74	11.85 ± 1.02	11.48 ± 1.02
Gall Bladder	4.33 ± 2.64	5.05 ± 3.56	2.83 ± 0.86	3.21 ± 1.02
Heart	2.22 ± 0.11	1.61 ± 0.06	1.43 ± 0.20	1.05 ± 0.05
Lung	2.25 ± 0.39	1.69 ± 0.07	1.33 ± 0.15	1.16 ± 0.22
Spleen	0.41 ± 0.13	0.31 ± 0.05	0.35 ± 0.05	0.38 ± 0.14
Stomach	18.07 ± 2.91	11.13 ± 1.16	6.40 ± 1.02	3.14 ± 0.62
Sm. Intestines	9.55 ± 1.21	4.58 ± 0.62	2.28 ± 0.10	1.24 ± 0.11
Lg. Intestines	9.83 ± 0.69	5.58 ± 0.94	3.95 ± 0.81	3.00 ± 0.37
Skin	2.72 ± 0.31	1.48 ± 0.03	1.22 ± 0.22	0.92 ± 0.14
Bone	0.59 ± 0.15	0.28 ± 0.21	0.24 ± 0.09	0.26 ± 0.01
Brain	0.12 ± 0.002	0.08 ± 0.01	0.09 ± 0.01	0.10 ± 0.02
Urine	25.10 ± 6.79	1.70 ± 1.91*	3.24 ± 1.03	1.46 ± 1.17
Fecal Matter	9.32 ± 1.08	4.58 ± 1.03	2.67 ± 0.58	2.29 ± 0.53

Biodistribution data for compound [^{64}Cu]**2** in mice bearing BxPC-3 xenograft tumors. Organ uptake is expressed as percent of injected dose per gram of tissue (% ID/g, *n* = 3, animals/ time point,*-only 2 mice provided urine).

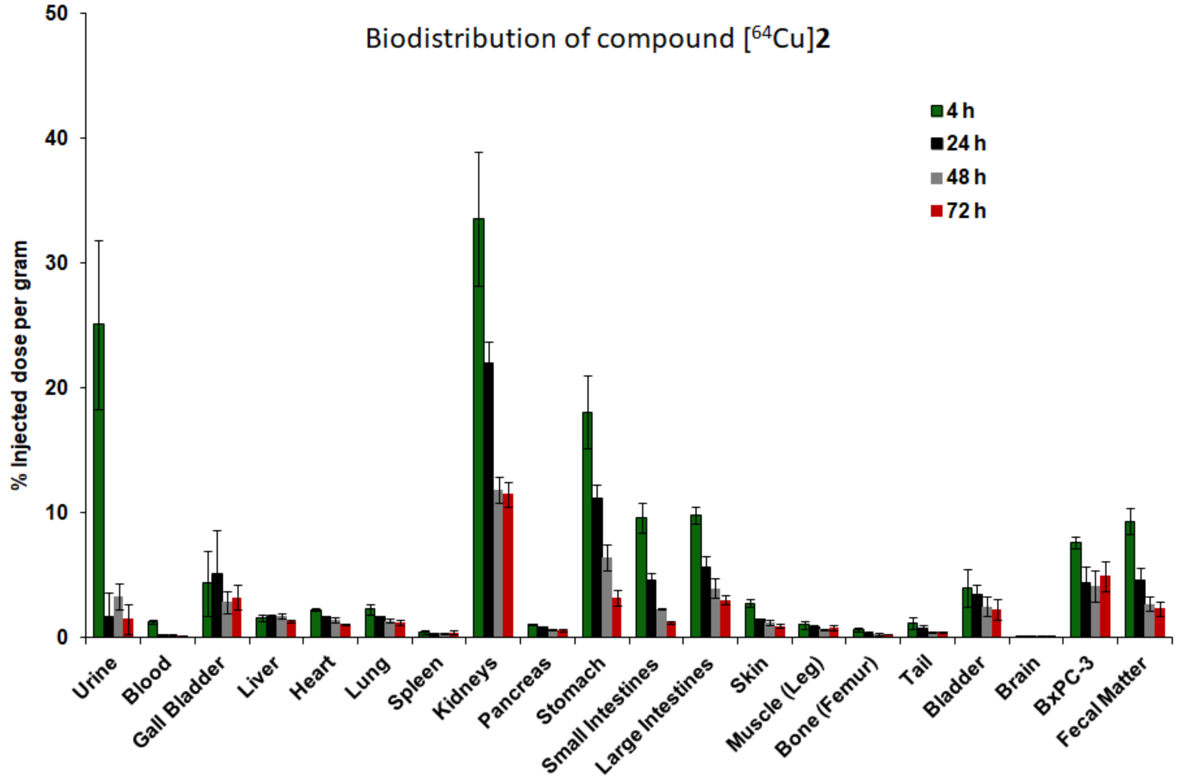

Figure S21. Biodistribution of [^{64}Cu]**2.**

Tumor-to-Organ ratios from 4 h to 72 h p.i. **A**. [^{64}Cu]**1** , **B**. [^{64}Cu]**2**

A.

**Tumor-to-Organ Ratios**([^{64}Cu]**1**)				
Time post-injection	4 h	24 h	48 h	72 h
Tumor-Blood	4.30±1.47	15.41±3.77	12.03±1.44	10.07±3.24
Tumor-Muscle	7.95±0.55	9.47±4.84	8.39±3.59	8.11±3.02
Tumor-Kidney	0.07±0.003	0.13±0.06	0.15±0.02	0.19±0.08
Tumor-Liver	3.24±0.14	2.39±0.59	1.51±0.29	1.47±0.47
Tumor-Stomach	0.83±0.03	1.19±0.02	1.41±0.79	1.65±0.32
Tumor-Sm Intestines	1.12±0.05	1.75±0.52	2.18±0.91	2.43±0.45
Tumor-Lg Intestines	1.28±0.12	1.41±0.43	1.51±0.58	1.75±0.48

B.

**Tumor-to-Organ Ratios**([^{64}Cu]**2**)				
Time post-injection	4 h	24 h	48 h	72 h
Tumor-Blood	6.15±0.47	21.86±5.94	22.11±14.41	28.82±2.64
Tumor-Muscle	8.16±2.41	5.87±2.04	6.68±2.42	6.70±1.74
Tumor-Kidney	0.23±0.02	0.20±0.06	0.35±0.14	0.44±0.14
Tumor-Liver	4.89±0.63	2.72±0.62	2.46±0.97	3.77±0.72
Tumor-Stomach	0.43±0.07	0.39±0.09	0.67±0.27	1.55±0.08
Tumor-Sm Intestines	0.80±0.07	0.97±0.27	1.81±0.61	4.01±1.10
Tumor-Lg Intestines	0.78±0.10	0.81±0.24	1.10±0.47	1.63±0.32

Figure S22. Tumor-to-organ ratios from 4 h to 72 h p.i. of [^{64}Cu]**1** and [^{64}Cu]**2.**

Blocking biodistribution of compounds [^{64}Cu]**1** & [^{64}Cu]**2** at 4 h p.i.

Tissue	[^{64}Cu]**1**	[^{64}Cu]**2**
BxPC-3 Tumor	2.91	2.89
Blood	2.56	3.98
Pancreas	0.39	0.76
Liver	1.92	2.04
Muscle	0.41	0.50
Kidneys	71.64	16.77
Gall Bladder	1.96	1.64
Heart	0.96	1.31
Lung	1.65	2.08
Spleen	0.85	0.00
Stomach	0.72	1.20
Sm. Intestines	0.91	1.18
Lg. Intestines	0.90	1.26
Skin	1.12	1.26
Bone	0.43	0.50
Brain	0.07	0.09

Blocking biodistribution data was obtained 4 h p.i. by blocking with ~220 nmol/kg (50 mg/kg) of compound DOTA-EB-$_{v}$ $_{6}$-BP (**1**) or DOTA-IP-$_{v}$ $_{6}$-BP (**2**) respectively, 10 minutes prior to the injection of either compound [^{64}Cu]**1** or [^{64}Cu]**2** in mice bearing BxPC-3 xenograft tumors. Organ uptake is expressed as percent of injected dose per gram of tissue (% ID/g, n = 1/compound at 4 h p.i.).

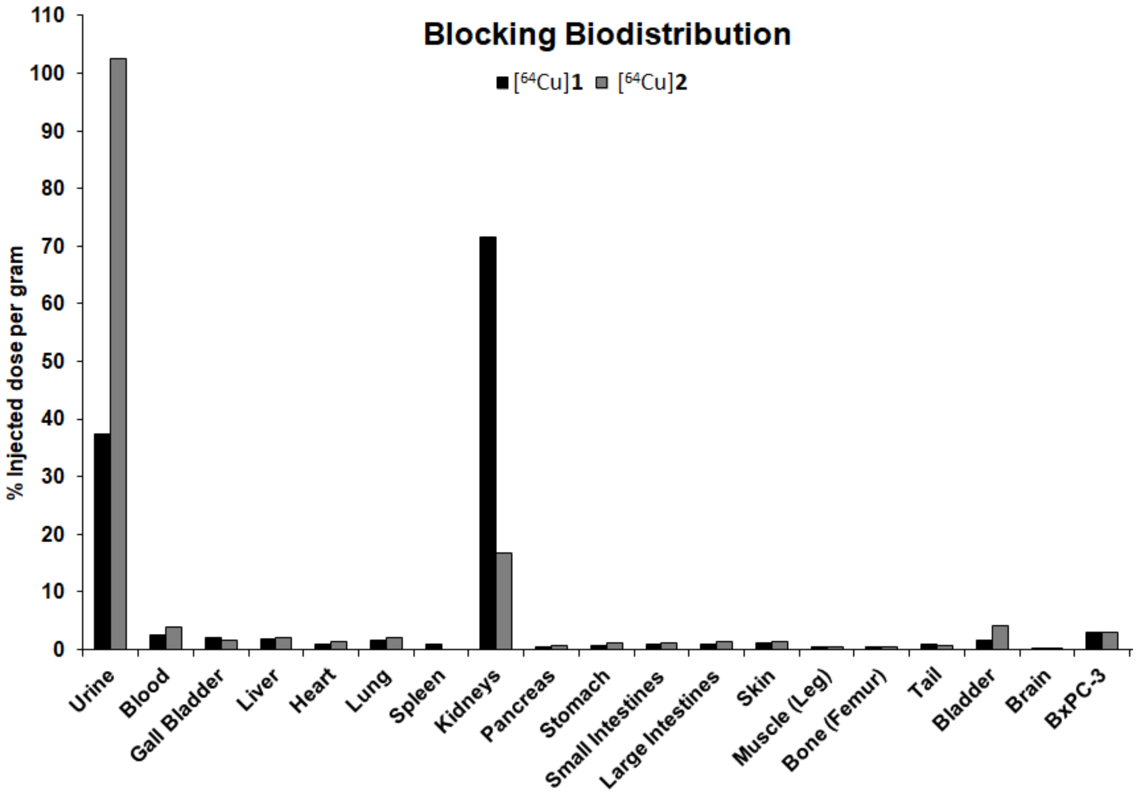

Figure S23. Blocking biodistribution of [^{64}Cu]**1** and [^{64}Cu]**2.**

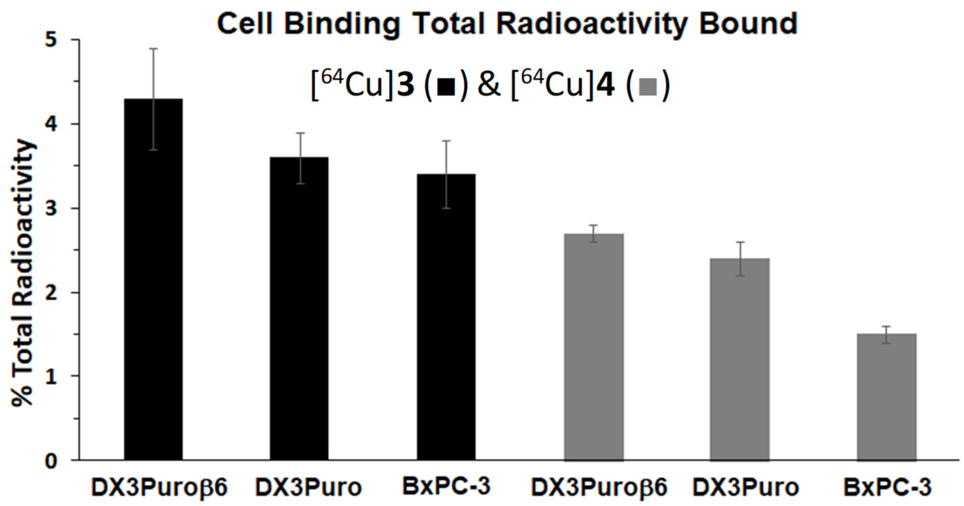

Figure S24. Cell binding assay of compounds [^{64}Cu]**3** & [^{64}Cu]**4**.

Biodistribution of compounds [64Cu]**3** & [64Cu]**4**

Tissue	[64Cu]**3**	[64Cu]**4**
BxPC-3 Tumor	24.82 ± 0.90	7.99 ± 1.93
Blood	38.88 ± 10.37	9.51 ± 1.32
Pancreas	6.40 ± 1.03	1.50 ± 0.09
Liver	22.01 ± 4.74	6.21 ± 1.51
Muscle	4.64 ± 1.40	1.28 ± 0.03
Kidneys	18.59 ± 1.41	4.34 ± 0.61
Gall Bladder	28.57 ± 10.67	10.23 ± 3.01
Heart	12.05 ± 2.69	3.07 ± 0.01
Lung	19.40 ± 1.33	5.01 ± 0.69
Spleen	8.10 ± 1.19	2.10 ±0.24
Stomach	10.16 ± 2.71	2.39 ± 0.31
Sm. Intestines	12.45 ± 3.06	3.53 ± 1.24
Lg. Intestines	12.75 ± 0.65	2.90 ± 1.05
Skin	14.77 ± 1.82	4.38 ± 0.77
Bone	4.81 ± 0.79	1.19 ± 0.06
Brain	0.97 ± 0.19	0.36 ± 0.05
Urine	63.25 ± 27.58	72.25 ± 35.13
Fecal Matter	33.79 ± 0.90	2.54 ± 1.41

Biodistribution data at 4 h post injection for compounds [64Cu]**3** &[64Cu]**4** in mice bearing BxPC-3 xenograft tumors. Organ uptake is expressed as percent of injected dose per gram of tissue (% ID/g, *n* = 2 animals/compound).

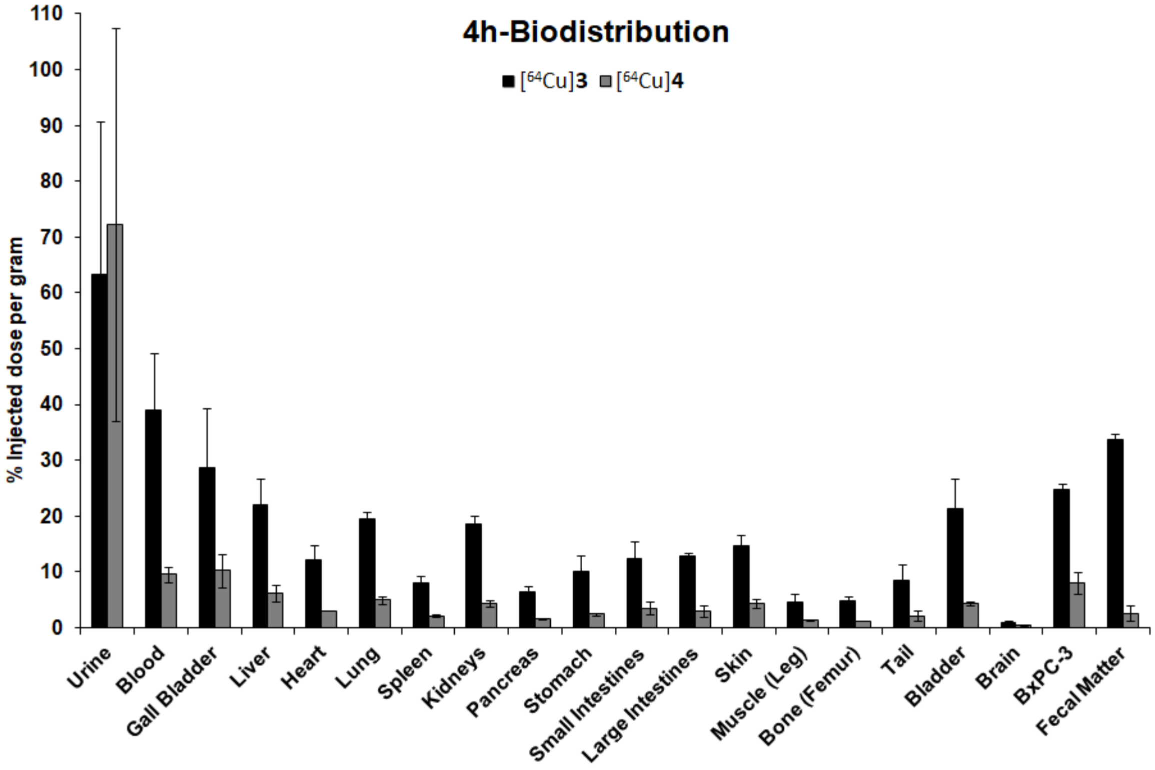

Figure S25. Biodistribution of [64Cu]**3** and [64Cu]**4.**

Summary of Tumor-to-Organ ratios at 4 h for [^{64}Cu]**1-4**

Tumor-to-Organ Ratios at 4 h				
Compounds:	[^{64}Cu]**1**	[^{64}Cu]**2**	[^{64}Cu]**3**	[^{64}Cu]**4**
Tumor-Blood	4.30±1.47	6.15±0.47	0.66±0.15	0.83±0.09
Tumor-Muscle	7.95±0.55	8.16±2.41	5.58±1.49	6.25±1.37
Tumor-Kidney	0.07±0.003	0.23±0.02	1.34±0.15	1.83±0.19
Tumor-Liver	3.24±0.14	4.89±0.63	1.15±0.21	1.29±0.001
Tumor-Stomach	0.83±0.03	0.43±0.07	2.52±0.58	3.31±0.37
Tumor-Sm Intestines	1.12±0.05	0.80±0.07	2.05±0.43	2.31±0.27
Tumor-Lg Intestines	1.28±0.12	0.78±0.10	1.49±0.68	2.83±0.36

Graph of Summarized Tumor-to-Organ ratios at 4 h for [^{64}Cu]**1-4**

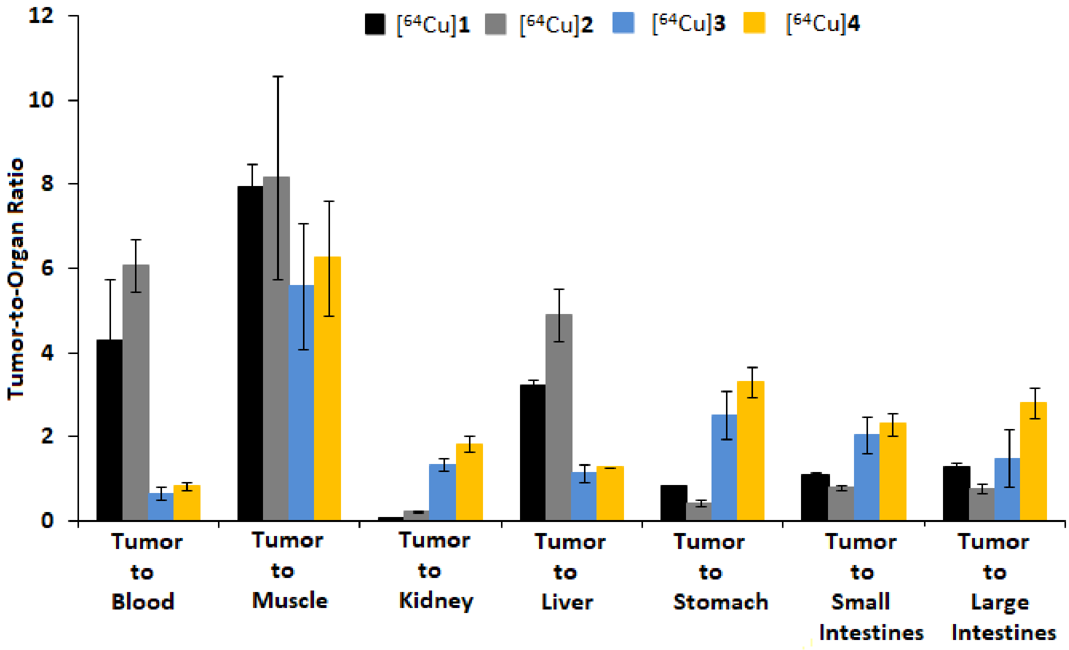

(**A**) Tumor-to-organ ratios at 4 h p.i. for [^{64}Cu]**1-4**. (**B**) Graph of tumor-to-organ ratios at 4 h p.i. for [^{64}Cu]**1** (■),[^{64}Cu]**2** (■),[^{64}Cu]**3** (■), and [^{64}Cu]**4** (■).

Figure S26. Summary of Tumor-to-organ ratios at 4 h for [^{64}Cu]**1–4.**
